# Oxidative Imbalance in Pediatric Anxiety Disorders: A Preliminary Comparative Study

**DOI:** 10.7759/cureus.54796

**Published:** 2024-02-23

**Authors:** Esen Yıldırım Demirdöğen, Çiğdem Tanrıverdi, İhsan Kara, Mehmet Ali Donbaloğlu, Fatma Betül Özgeriş

**Affiliations:** 1 Department of Child and Adolescent Psychiatry, Atatürk University Faculty of Medicine, Erzurum, TUR; 2 Department of Child and Adolescent Psychiatry, Erzurum Regional Training and Research Hospital, Erzurum, TUR; 3 Department of Nutrition and Dietetics, Faculty of Health Sciences, Ataturk University, Erzurum, TUR

**Keywords:** oxidative imbalance, oxidative stress index, total antioxidant status, total oxidant status, childhood anxiety disorder

## Abstract

Background

It is important to determine the possible related factors of anxiety disorder, one of the common psychiatric disorders of childhood. Our aims in this study were to compare oxidative stress markers between anxiety disorders in pediatric patients and healthy controls and to examine the relationship between anxiety symptom severity and oxidative stress indicators.

Methods

The study included 25 patients and 25 healthy controls. We measured the total oxidant capacity (TOS) and total antioxidant capacity (TAS) from the collected serum samples and calculated the oxidative stress index (OSI). We evaluated the clinical severity of the anxiety symptoms by the Revised Child Anxiety and Depression Scale-Child Version (RCADS-CV).

Results

The groups did not exhibit a noteworthy distinction in terms of TOS (p=0.128) and TAS (p=0.329). However, OSI was markedly elevated in the group with anxiety disorder (p=0.044). In the correlation analysis between anxiety symptom severity and oxidative stress indicators in the group with anxiety disorder, we found a positive correlation between TOS and RCADS total anxiety score (p=0.08).

Conclusion

These results may point to an oxidative dysfunction in anxiety disorders and the potential role of oxidative stress in their aetiology. Prospective, large-scale, randomized studies are needed to investigate if oxidative stress indicators can be used in the diagnosis of anxiety disorders and as new treatment targets.

## Introduction

Among pediatric and adolescent psychiatric disorders, anxiety disorders are a frequently encountered factor that adversely affects school performance, social relationships, overall health, and quality of life [1,2]. Despite anxiety disorders being prevalent in childhood psychiatry, their pathophysiology remains not fully understood. To explain the pathogenesis of anxiety disorders, researchers have suggested various psychological, behavioural, neurocognitive, and genetic-biological theories [3]. Recent research suggests that oxidative stress may have a pathophysiological role in anxiety disorders [4].

Free radicals are formed in the organism as a result of the reactions of oxygen in routine metabolic processes. All of the resulting free radicals combine to form oxidative stress. Oxidative stress has cytotoxic and genotoxic effects. For this reason, balancing oxidative stress by the antioxidant system and neutralization of oxidative stress components are necessary for the organism to continue its life. Oxidative damage occurs when oxidative stress increases and the antioxidant system cannot compensate for this situation [5-7].

The number of studies on the impact of oxidative stress on the etiopathogenesis of anxiety disorders in adults is on the rise [8-10]. However, research focusing on the effect of oxidative stress in anxiety disorders among children and adolescents is more limited [11]. In these studies, it has been suggested that complex mechanisms such as imbalance of neurotransmitters, hypothalamic-pituitary-renal axis, neuroinflammation and mitochondrial dysfunction may play a role between anxiety disorders and oxidative stress and that the basis of anxiety disorders can be better explained by examining these mechanisms in detail [8-11].

Many parameters are indicators of oxidative stress and antioxidant status. Because it would be difficult to measure and evaluate all parameters simultaneously, researchers have started a search for laboratory parameters that may be indicative of the overall oxidant-antioxidant status. Total oxidant capacity (TOS) and total antioxidant capacity (TAS) measurements were possible with the developed methods. The measurement of TOS indicates the overall oxidant status of the sample, whereas TAS measurement reflects the total antioxidant status of the sample. Researchers have demonstrated the measurement accuracy and validity of these parameters, and companies have begun commercial kit production for these measurements. In addition, the oxidative stress index (OSI) can be determined based on these two parameters. The importance of the OSI is that it shows the overall balance of oxidative stress and antioxidant status in a two-way manner [12,13].

In this context, our objective was to enhance the existing literature by comparing TOS, TAS, and OSI levels between anxiety disorders in pediatric patients and healthy controls. Additionally, we aimed to investigate the association between the severity of anxiety symptoms and these indicators of oxidative stress. Our study stands as one of the few instances in the literature to concern TOS and TAS levels in anxiety disorders among children and adolescents. To our knowledge, it represents the first clinical study to offer great detail on the relationship between anxiety symptom severity and these oxidative stress indicators. Our hypothesis was that TOS levels and OSI levels would be higher and TAS levels would be lower in children with anxiety disorders than in healthy controls, and these parameters would be related to anxiety symptom severity.

## Materials and methods

Study design and participants

We initiated the study with the approval of the local ethical committee (IRB approval no: B.30.2.ATA.0.01.00/419). In the anxiety disorder group, six children were excluded; because three children had psychiatric comorbid diagnoses (two with attention deficit hyperactivity disorder, one with obsessive-compulsive disorder), one child had epilepsy, and one child had type 1 diabetes mellitus, and the family of one participant did not consent to participate in the study. In the control group, four children were excluded from the study because two children had a psychiatric diagnosis (one with attention deficit hyperactivity disorder and one with major depressive disorder) and two children had a diagnosis of chronic medical illness (obesity and familial Mediterranean fever). Consequently, the study included 25 patients diagnosed with anxiety disorders for the first time who we evaluated at the outpatient clinic of our hospital and 25 healthy children and adolescents who applied to pediatric outpatient clinics for routine controls within the same period. The anxiety disorder diagnosis was determined by a clinical assessment based on the Diagnostic and Statistical Manual of Mental Disorders-5 (DSM-5). To confirm the diagnosis and exclude comorbid psychiatric disorder, a semi-structured diagnostic interview (Kiddie Schedule for Affective Disorders and Schizophrenia for School-Age Children - Present and Lifetime Version - Turkish Version; KSADS-PL-T) was conducted. All participants were aged 8-17 years. We excluded patients with a history of comorbid psychiatric, neurologic, genetic, endocrinologic, allergic, or chronic systemic diseases; history of alcohol and cigarette smoking; and previous use of psychotropic drugs. In the control group, in addition to the aforementioned exclusion criteria, having a psychiatric disorder was also an exclusion criterion. We obtained written informed consent from both groups from all patients and their parents.

Scales

We employed the KSADS-PL-T in our study. K-SADS-PL is a diagnostic interview specially designed to evaluate current and past psychiatric diagnoses in pediatric patients, and its Turkish validity and reliability were established by Ünal [14,15]. In our study, it served the purpose of confirming the diagnosis and ruling out comorbidities in the case group, as well as excluding any psychiatric diagnoses in the control group.

We also utilized the Revised Child Anxiety and Depression Scale - Child Version (RCADS) in our study. The RCADS encompasses subscales in anxiety disorders. Additionally, the scale offers an overall anxiety score, which is the sum of scores on all anxiety subscales [16]. Görmez et al. conducted the Turkish validity and reliability study for RCADS in the 8-17 age group [17].

Blood sample collection and analysis

Following an overnight fasting period, we drew around 10 ml of blood from the antecubital vein of each participant between 8:00 a.m. and 10:00 a.m. for biochemical analysis. We placed the blood taken from the patients in serum tubes containing gel reagents. Subsequently, we extracted serum through centrifugation at 3000 rpm for 10 minutes. We kept serum samples obtained by centrifugation at -80°C until the day of analysis. According to the method developed by Erel, we measured TOS levels by an automatic colorimetric method [13]. We quantified and reported values in micromoles of hydrogen peroxide equivalents per liter (μmol H2O2 Equiv/L). We determined TAS levels using an autoanalyzer, employing the method developed by Erel [12]. During measurement of TAS level, ferrous (2+)-o-dianisidine forms hydroxyl radicals through a Fenton-type chemical interaction with hydrogen peroxide. These highly reactive oxygen species undergo reduction and chemically react with o-dianisidine at low pH, resulting in the formation of brown-yellow dianisidyl radicals. These radicals actively engage in oxidation reactions, augmenting the formation of color. Conversely, antioxidants present in the samples mitigate coloration by impeding these oxidation-reduction reactions. We determined the TAS value with this decrease detected spectrophotometrically. The unit employed was mmol Trolox Equiv/L. We calculated OSI results using the formula [(TOS, μmol H2O2 Equiv/L)/(TAS, mmol Trolox Equiv/L)/100] [12,13].

Statistical analysis

We conducted all statistical analyses utilizing the Statistical Package for the Social Sciences (IBM SPSS Statistics for Windows, IBM Corp., Version 22.0, Armonk, NY). We employed the Kolmogorov-Smirnov test to assess statistical normal distribution. We used numbers and percentages for categorical variables. We used means and standard deviations for continuous variables. We utilized the Chi-square test for comparing categorical variables. We assessed numerical variables using independent samples t-test and Mann-Whitney U test, considering their normal distribution. We applied Spearman correlation analysis to evaluate the relationship between oxidative stress indicators and anxiety severity.

## Results

The study encompassed 25 patients and 25 healthy controls. The mean ages of the patient group and control group were 13.4±2.73 and 13.2±2.76 years, respectively. It was statistically similar in terms of age between both groups (p = 0.798). Gender distribution was also statistically similar between the groups (p = 0.333). Of the children with anxiety disorders, 11 (44%) had generalized anxiety disorder, five (20%) had social anxiety disorder, four (16%) had separation anxiety disorder, two (8%) had panic disorder, and three (12%) had specific phobia. We share the characteristics of those included in the study in detail in Table 1.

**Table 1:** The sociodemographic data of the sample Lowercase letters are used to indicate the statistical tests used in Table 1. a: Student's t-test b: Chi-square test c: Fisher's exact test

	Anxiety Disorder Group n=25	Control Group n=25	t/Z/X^2 ^	p-value
Age	13.4 ± 2.73	13.2 ± 2.76	0.257	0.798^a^
Gender			0.936	0.333^b^
Girl	20 (80%)	17 (68%)		
Boy	5 (20%)	8 (32%)		
Mothers’ education (year)	9.73±3.25	10.51±4.42	0.182	0.420^a^
Fathers’s education (year)	10.65±3.01	11.24±2.34	0.715	0.632^a^
Family income			0.951	0.672^c^
Low	4 (16%)	6 (24%)		
Middle	10 (40%)	11 (44%)		
High	11 (44%)	8 (32%)		
Family type			5.717	0.089^c^
Nuclear family	21 (84%)	16 (64%)		
Extended family	1 (4%)	7 (28%)		
Fragmented family	3 (12%)	2 (8%)		
Family history of psychiatric disorder			0.104	0.747^b^
Yes	7 (28%)	6 (24%)		
No	18 (72%)	19 (76%)		
Generalized Anxiety Disorder	11 (44%)	-		
Social Anxiety Disorder	5 (20%)	-		
Separation Anxiety Disorder	4 (16%)	-		
Panic Disorder	2 (8%)	-		
Specific Phobia	3 (12%)	-		

**Figure 1:**
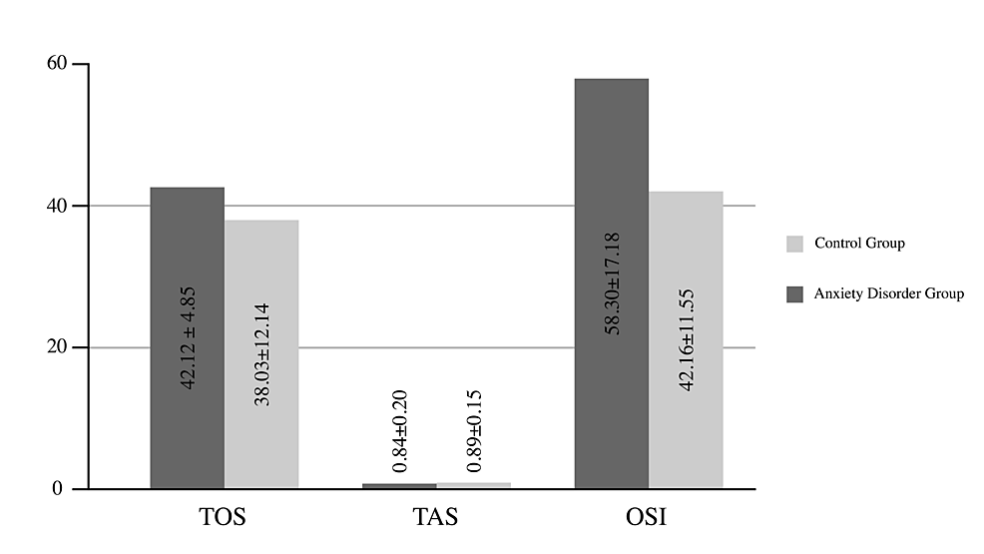
Serum TOS, TAS, and OSI levels TOS: Total oxidant status, TAS: Total antioxidant status, OSI: Oxidative stress index

In the anxiety disorder group, TOS, TAS, and OSI values were (42.12±4.85, 0.84±0.20, 58.30±17.18), respectively. In the control group, TOS, TAS, and OSI values were (38.03±12.14, 0.89±0.15, 42.16±11.55), respectively. TOS and TAS levels were statistically similar for both groups (t=1.562, p=0.128 for TOS and t=-0.987, p=0.329 for TAS). However, OSI was statistically significantly lower in the control group (t=2.072, p=0.044). We summarize oxidative stress indicator data and comparisons of both groups in Figure 1.

In the correlation analysis examining the relationship between anxiety symptom severity and oxidative stress indicators within the anxiety disorder group, we observed a positive correlation between the TOS and scores on the panic disorder subscale (r=0.456, p=0.022), separation anxiety disorder subscale (r=0.417, p=0.038), generalized anxiety disorder subscale (r=0.397, p=0.049), and total anxiety score (r=0.518, p=0.008). The correlation analysis results are presented in Table 2.

**Table 2:** Correlations between TOS, TAS, OSI levels, and children's anxiety scores TOS: Total oxidant status, TAS: Total antioxidant status, OSI: Oxidative stress index, r: The Pearson correlation coefficient

		Generalized Anxiety	Social Anxiety	Separation Anxiety	Panic	Total Anxiety	Major Depressive Disorder	Obsessive Compulsive Disorder
TOS	r	0.397	0.305	0.417	0.456	0.518	0.377	0.153
	p	0.049	0.138	0.038	0.022	0.008	0.064	0.466
TAS	r	0.237	-0.068	0.257	0.066	0.170	0.267	0.212
	p	0.254	0.748	0.215	0.754	0.416	0.197	0.309
OSI	r	-0.211	0.006	-0.199	-0.148	-0.190	-0.275	-0.199
	p	0.312	0.976	0.340	0.479	0.362	0.184	0.339

## Discussion

Numerous antioxidant and oxidant molecules serve as indicators of the oxidative stress state within the body, yet individually measuring their levels is challenging and costly. Therefore, practical measurements such as TOS and TAS levels are employed. TAS reflects the cumulative effect of all antioxidants, whereas TOS signifies the collective impact of all oxidants present in body fluids [18]. In humans, a delicate equilibrium exists between the production and elimination rates of oxidants and antioxidants, safeguarding the body against the detrimental effects of free oxygen radicals. Oxidative stress arises when there is an imbalance between free radicals and the antioxidant defenses tasked with counteracting their effects. OSI, as a measure of oxidative stress, is computed through the ratio of TOS to TAS [19].

Approximately 20% of the oxygen in the body is used by the brain [20]. Therefore, significant amounts of free oxygen radicals are produced in the brain. In addition, the human brain contains large amounts of fatty acid compounds that facilitate oxidation [7]. When free oxygen radicals rise above physiological levels, vital molecules can be damaged [21]. Research has demonstrated that the brain is particularly vulnerable to oxidative stress, given its elevated oxygen demand and abundant polyunsaturated fatty acids [22]. There is accumulating evidence supporting the significant involvement of oxidative stress in the pathophysiology of psychiatric disorders [23]. Numerous studies have indicated that the dysregulation between free oxygen radicals and the antioxidant system is implicated in the pathogenesis of nervous tissues, correlating with various neurological and psychiatric disorders [24]. Substantial progress has been achieved in recent years in comprehending the phenomenology, genetics, and neurobiology of pediatric anxiety disorders [25]. Although the cause-and-effect relationship between these disorders and oxidative stress is not clear, it is thought that oxidative stress is thought to be involved in anxiety disorders by affecting systems that regulate anxiety. Studies in both adulthood and childhood have explained the relationship between oxidative stress and anxiety disorders through various mechanisms: Oxidative stress can affect the balance of neurotransmitters (e.g. serotonin, dopamine, norepinephrine) by damaging the lipid membranes of nerve cells. This can lead to imbalances in nerve transmission and anxiety disorders. The Hypothalamus-pituitary-renal (H-P-A) axis, which is involved in the regulation of the stress response, can be affected by oxidative stress. Increased release of stress hormones such as cortisol can increase oxidative stress, which in turn can lead to anxiety symptoms. Oxidative stress can trigger or enhance inflammatory processes in the brain. This can enhance the activation of microglial cells and increase the release of proinflammatory cytokines (e.g. interleukin-1β, tumor necrosis factor-alpha). Neuroinflammation may play an important role in the pathogenesis of anxiety disorders. Oxidative stress can lead to mitochondrial dysfunction and cellular damage. This, in turn, can affect the functionality of neurons and lead to anxiety symptoms [8,11]. In addition, studies suggest that correcting oxidative imbalance in psychiatric disorders may have therapeutic effects independent of the underlying mechanisms [26].

Several researchers have explored the role of oxidative stress, oxidative stress markers, and antioxidant levels in anxiety disorders [27]. Notably, these authors have primarily focused their investigations on adults. For instance, a study of oxidative stress markers in adults with generalized anxiety disorder revealed significantly elevated TOS and OSI levels in patients compared to the control group, with TAS levels being significantly lower in the patient group [9]. In another study to evaluate TAS and TOS levels in individuals with panic disorder, the authors reported significant differences in oxidative stress markers compared to healthy groups [7].

Despite the abundance of studies in adults, research on oxidative stress markers in anxiety disorders in pediatric patients remains limited. In one notable study, Güney et al. compared TAS, TOS, and OSI levels in groups of 40 patients with anxiety disorders and 35 healthy subjects. The authors found significantly higher TOS and OSI levels in the anxiety disorder group compared to the control group. However, they acknowledged the limitation of not assessing the relationship between anxiety severity and oxidative stress parameters in their study [11].

In our study, we examined the oxidative stress markers TAS, TOS, and OSI in patients diagnosed with anxiety disorders and a healthy control group. Our findings revealed a statistically significant elevation in OSI levels in the anxiety disorder group. Although TOS and TAS levels did not exhibit a statistically significant difference, TOS was relatively high, and TAS was relatively low in the patient group. These results suggest an increased oxidant load and an impaired balance between oxidant/antioxidant mechanisms in patients with anxiety disorders. Our findings align with other studies in the literature concerning oxidative stress markers in patients with anxiety disorders.

A noteworthy aspect of our study is the examination of the relationship between anxiety severity and oxidative stress markers in children and adolescents. We identified a positive correlation between TOS and separation anxiety disorder subscale score, generalized anxiety disorder subscale score, and total anxiety score. Based on our results, we posit that oxidative stress warrants further investigation for a comprehensive understanding of clinical features in patients with anxiety disorders, serving as a guide for researchers interested in this subject.

Our study has some limitations. One of these is the small sample size. The small sample size limits the generalizability of our results. Furthermore, the conceptual validity of the study may also be affected by the small sample size. These results, based on a small sample, may raise questions about whether the measures and methods used are fully representative of the general population with anxiety disorders. A larger and more diverse group of participants is needed to understand a complex condition such as anxiety disorders. Another important limitation is the wide age range of the children included in the study. Due to the wide age range, our results may have been affected by the variability in the parameters with age. In addition, the cross-sectional design of the study prevents the establishment of a full cause-effect relationship between the results. Therefore, it is not possible to talk about a cause-effect relationship in anxiety disorder related to these parameters. Finally, the heterogeneity of the sample, particularly in terms of anxiety disorder subtypes, represents a significant limitation. Due to this situation, we could not make any comments on anxiety disorder subtypes.

## Conclusions

Despite these limitations, our study stands as one of the few instances in the literature that constitutes an exploration of the role of oxidative stress in anxiety disorders in pediatric patients. Moreover, to the best of our knowledge, it is the first clinical study whose authors investigate the relationship between anxiety symptom severity and oxidative stress indicators in this demographic.

These results may point to an oxidative dysfunction in anxiety disorders and the potential role of oxidative stress in their etiology. Prospective, large-scale, randomized studies are needed to investigate if oxidative stress indicators can be used in the diagnosis of anxiety disorders and as new treatment targets.
